# Effect of Cryogenic Treatments on Hardness, Fracture Toughness, and Wear Properties of Vanadis 6 Tool Steel

**DOI:** 10.3390/ma17071688

**Published:** 2024-04-07

**Authors:** Venu Yarasu, Peter Jurci, Jana Ptacinova, Ivo Dlouhy, Jakub Hornik

**Affiliations:** 1 Faculty of Materials Technology Based in Trnava, Slovak University of Technology Based in Bratislava, 917 24 Trnava, Slovakia; venu.yarasu@gmail.com (V.Y.); jana.ptacinova@stuba.sk (J.P.); 2 Institute of Physics of Materials, Czech Academy of Sciences, Zizkova 22, 616 62 Brno, Czech Republic; idlouhy@ipm.cz; 3 Department of Materials Engineering, Faculty of Mechanical Engineering, Czech Technical University, 160 00 Prague, Czech Republic; jakub.hornik@fs.cvut.cz

**Keywords:** cryogenic treatment, cold-work tool steel, hardness, fracture toughness, tribological properties, wear mechanisms

## Abstract

The ability of cryogenic treatment to improve tool steel performance is well established; however, the selection of optimal heat treatment is pivotal for cost reduction and extended tool life. This investigation delves into the influence of distinct cryogenic and tempering treatments on the hardness, fracture toughness, and tribological properties of Vanadis 6 tool steel. Emphasis was given to comprehending wear mechanisms, wear mode identification, volume loss estimation, and detailed characterization of worn surfaces through scanning electron microscopy coupled with energy dispersive spectroscopy and confocal microscopy. The findings reveal an 8–9% increase and a 3% decrease in hardness with cryogenic treatment compared to conventional treatment when tempered at 170 °C and 530 °C, respectively. Cryotreated specimens exhibit an average of 15% improved fracture toughness after tempering at 530 °C compared to conventional treatment. Notably, cryogenic treatment at −140 °C emerges as the optimum temperature for enhanced wear performance in both low- and high-temperature tempering scenarios. The identified wear mechanisms range from tribo-oxidative at lower contacting conditions to severe delaminative wear at intense contacting conditions. These results align with microstructural features, emphasizing the optimal combination of reduced retained austenite and the highest carbide population density observed in −140 °C cryogenically treated steel.

## 1. Introduction

Tool steels play a crucial role in various industrial applications, where their performance and longevity hinge on the delicate balance of microstructural characteristics and mechanical properties. Particularly in operations such as blanking, stamping, and punching, where stringent requirements for high hardness, fracture toughness, and resistance to abrasive wear are paramount, the choice of heat treatment method significantly influences the material’s suitability for these tasks. However, conventional heat treatment techniques often encounter performance plateaus, constraining their efficacy and adaptability to demanding environments.

In response to these limitations, cryogenic treatment has emerged as a promising supplementary technique for enhancing the properties of tool steels across various categories, including cold-work [1,2,3], hot-work [4,5], high-speed [6,7], and high-strength steels [8,9]. Cryogenic treatment, typically performed between hardening and tempering stages, involves subjecting the material to extremely low temperatures ranging from −60 to −196 °C, followed by controlled reheating to ambient conditions. This process induces significant microstructural modifications in tool steels, which have been extensively studied and documented in the literature [1,2,3,4,5,6,7,8,9].

Studies have consistently reported that cryogenic treatment induces beneficial alterations in the microstructure of tool steels, resulting in increased hardness and improved wear resistance. These improvements are attributed to several factors. The majority of the studies linked the enhanced performance to the lowering of retained austenite (γ_R_) and the enhanced carbide populations [1,2,3,4,5,6,7,8,9,10]. Other studies also highlighted the refinement of martensite with a greater number of dislocation densities and fine twining, the formation of secondary carbides, and the establishment of a more homogeneous distribution of nano-sized transient precipitates [6,8,11,12]. However, the specific mechanisms driving these enhancements and their extent vary depending on the material composition and processing parameters.

For instance, Guili Xu et al. [6] investigated the impact of the duration of cryogenic treatment (in the range of 3 min–48 h) on the mechanical properties of AISI M35 high-speed steel in both un-tempered and tempered states. They observed that the hardness (HV1) of cryogenic treatment (CT) specimens increased by 44 HV1 (5.1% increase) when treated for 5 h compared with conventionally treated specimens. Additionally, they reported that treatment duration had only a marginal effect on hardness when it exceeded 5 h. Moreover, the impact toughness increased by 32.4%, and the hardness increased by only 2.6% of CT specimens when treated for 5 h and tempered over quenched and tempered specimens.

Similarly, Dhokey et al. [10] explored the impact of cryogenic treatment on wear performance in AISI D2 cold-work tool steel, highlighting substantial improvements in hardness and wear resistance, albeit with some compromise in impact toughness. They reported a maximum hardness of 62.74 HRC (6.8% increase) and a maximum wear resistance index of 4085 (252% increase) over conventional treatment after tempering at 450 °C, followed by cryogenic treatment with single tempering at 250 °C for 24 h. However, they noted a 23.2% reduction in impact toughness compared to conventional treatment. Similar findings were reported by other researchers [1]. They observed an average increase of 1.5% in hardness but a decrease of 10.7% in fracture toughness for AISI D2 steel when treated at −196 °C for 4 h, followed by single tempering at 480 °C.

In addition to the aforementioned studies, further investigations by other research groups have provided valuable insights into the effects of cryogenic treatment (CT) on the wear resistance of AISI D2 steel. For instance, research studies conducted in the literature [13,14,15,16] explored the impact of CT at temperatures ranging from −80 °C to −196 °C, with treatment durations spanning from 0 to 132 h. The findings revealed that treatment at −196 °C for 32 h yielded the highest wear resistance, resulting in a remarkable 69% improvement over conventional treatment. Similarly, Amini et al. [17] reported significant enhancements in wear resistance, demonstrating a 60–66% improvement following treatment at −196 °C for 48 h compared to conventional methods. Furthermore, Korade et al. [18] investigated various heat treatment strategies, including hardening, tempering cycles, and cryogenic treatment at −185 °C for 36 h, on the wear rate of AISI D2 steel. Their results indicated that a combination of hardening, CT, and single tempering led to a notable 5% increase in hardness and a substantial 59.74% reduction in wear rate compared to conventional treatment. Additionally, CT at −140 °C for 2 h exhibited a remarkable 50.7% improvement in wear performance over conventionally heat-treated (CHT) steel [19]. These studies collectively underscore the significant potential of cryogenic treatment in enhancing the wear resistance of D2 cold-work tool steel, offering valuable guidance for optimizing heat treatment strategies in industrial applications.

Despite the considerable body of research elucidating the benefits of cryogenic treatment, there remains a need for comprehensive studies focusing on specific tool steel grades and optimizing process parameters to maximize performance. In this context, the present study addresses the optimization of performance in Vanadis 6 steel, a cold-work tool steel widely utilized in industrial applications such as blanking and fine blanking of harder work materials, powder compacting, manufacturing of plastic molds, knives, and others. The primary objective of this study is twofold: first, to identify the optimal combination of process conditions for enhancing the hardness, fracture toughness, and tribological performance of Vanadis 6 steel, and second, to elucidate the underlying wear mechanisms under various tribological conditions. To achieve these goals, a combination of statistical analyses and microstructural examinations were employed.

## 2. Materials and Methods

### 2.1. Materials

In this study, Vanadis 6 plate-like steel samples (for wear testing) with dimensions of 30 × 30 × 6 mm and 10 × 10 × 55 mm specimens (for fracture toughness determination) were used with a nominal (given by the steel manufacturer) and actual (measured by spectrometer) composition, as presented in Table 1.

**Figure 1:**
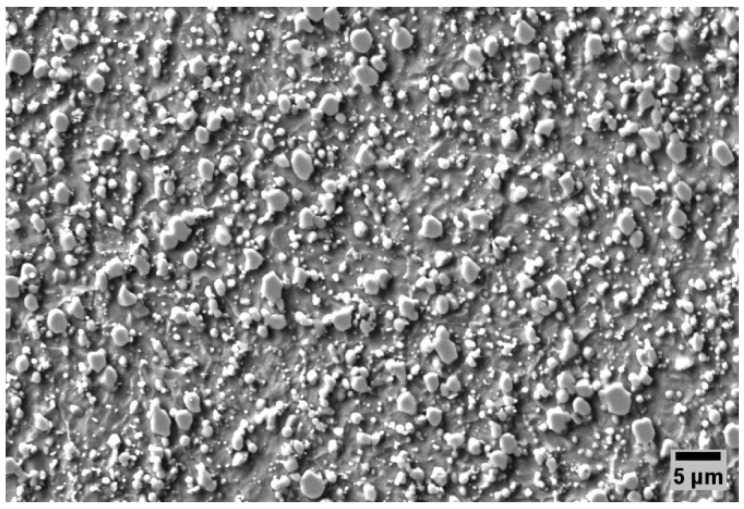
SEM microstructure observation of Vanadis 6 steel in soft-annealed state.

The as-received (annealed) microstructure of Vanadis 6 PM steel contains the matrix (ferrite) and spheroidized carbides, as shown in Figure 1. The initial (as-received) hardness was 278 HV10.

**Figure 2:**
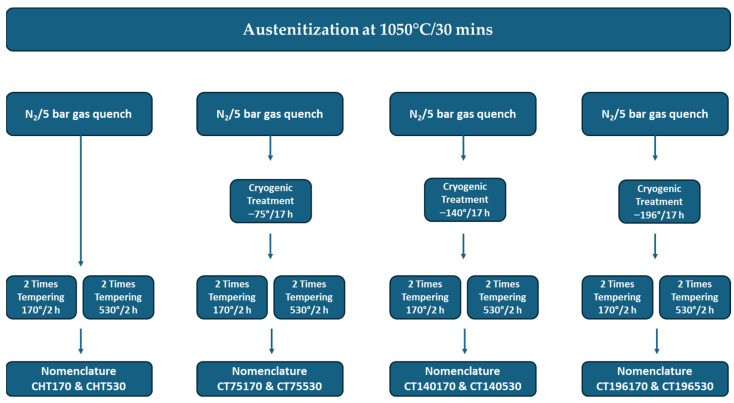
Heat treatment cycles applied to Vanadis 6 steel.

The samples were subjected to vacuum heat treatment that consisted of heating to the austenitizing temperature (1050 °C), holding at this temperature for 30 min, and nitrogen gas quenching (5 bar). The detailed heat treatments used in this study are shown in Figure 2.

In the cryogenic treatment process, quenched samples were further cooled to different cryogenic temperatures (−75, −140, and −196 °C) using a controlled rate of 1 °C per minute. They were held at these temperatures for 17 h before being slowly warmed back to room temperature at the same rate. Finally, the samples were subjected to two separate tempering treatments (Tps): either at 170 °C (low temperature tempering) or at 530 °C (high temperature tempering). Each tempering treatment (Tp) lasted for 2 h. The choice of cryogenic treatment parameters was based on recent investigations [20,21,22], where temperatures of −75 °C, −140 °C, and −196 °C were used, and a duration of 17 h was found to achieve the best combination of retained austenite reduction and the highest population density of extra carbides.

### 2.2. Methods

For microstructural observations, the samples were prepared using standard metallographic methods, which include grinding, polishing, and etching with Villela-Bain reagent (a solution of 1 g of picric acid, 5 mL of hydrochloric acid, and 100 mL of ethanol). Microstructural features were observed using a scanning electron microscope (SEM) JSM-7600F (Jeol Ltd., Tokyo, Japan). Microstructural observations were coupled with energy-dispersive spectroscopy (EDS) (Oxford Instruments, plc., High Wycombe, UK) wherever necessary. Quantitative analysis of carbides was conducted on randomly acquired high-magnification twenty SEM images using Image J software.

Vickers hardness tests were conducted, adhering to the ASTM E384 standard, to assess the samples hardness. Each sample received a 10 kgf (98 N) indentation force applied for 15 s. Five distinct locations on each sample were measured to obtain an accurate average hardness value. Furthermore, fracture toughness (K_IC_) tests were conducted on pre-cracked, heat-treated specimens. Both pre-cracking and the tests were carried out at room temperature, according to the ISO 12137 standard [23]. For the determination of fracture toughness, the samples were loaded in three-point bending with a roller span of 40 mm and a loading rate of 0.1 mm·min^−1^. Five samples were tested for each investigated condition, to ensure the statistical reliability of the obtained data.

Dry sliding wear tests were carried out by using a CSM pin-on-disk apparatus in accordance with the ASTM G99 standard [24]. Sintered alumina balls, 6 mm in diameter and with an estimated microhardness of 2450 HV0.1, were used as counterparts. The measurements were performed at room temperature (24 °C) with normal loads of 1, 5, or 10 N and three sliding velocities, namely, 0.064, 0.128, and 0.1885 m/s. The total sliding distance was 100 m. The wear volume loss was calculated using Equation (1) [24], where V is the volume loss in mm^3^, R is the wear track radius (in mm), d is the mean width of the wear track (in mm), and r is the radius of the counterpart ball (in mm). Three trials were performed at each combination of processing parameters and testing conditions, and the average values of the obtained results were then calculated.

V = 2ΠR [r^2^/sin(d/2r)] − [(d/4) √(4r^2^ − d^2^)] (1)

Furthermore, the Taguchi optimization technique was used to conduct the experiments, taking into account the design factors of cryogenic temperature, tempering temperature, sliding velocity, and load. The Taguchi design has gained great popularity over the past decade since it enables reducing the number of trials to estimate the impact of processing parameters on the resulting product characteristics [25]. To achieve optimal quality performance, the Taguchi method employs signal-to-noise ratio (SN) analysis for optimizing process settings.

This paper utilized Taguchi’s L18 orthogonal array, involving 18 experiments, to conduct tribological tests. The results are reported as average values estimated from the three identical tribological tests. Taguchi’s design is a good tool that allows for the determination of material quality characteristics based on input and output conditions’ means and signal-to-noise (SN) ratios. The ideal outcome is mainly influenced by the SN ratio’s effect on the response. Taguchi design recognizes different categories of desirable outcomes, such as minimum better, maximum better, and nominal better. This study focused on minimizing the coefficient of friction (COF) and volume loss (V), as these are key indicators of performance. Lower values of both metrics represent better performance, so minimum is a better attribute, and Equation (2) [25] was used in this study, which considers the number of trials (n) in the factor level combination and the responses (y) for the given factor level combination. The SN ratio for each parameter was then used to determine the best processing parameters, with a higher SN ratio corresponding to a lower response value (i.e., friction coefficient and volume loss). Finally, an analysis of variance (ANOVA) test was performed using MINITAB 19 software with a 95% confidence interval and 5% significance level to determine the contribution of individual factors to the response variable.

Minimum is better (S/N) = −10 log (∑y^2^/n) (2)

The obtained worn surfaces were studied using a SEM coupled with EDS. For a qualitative and quantitative analysis of wear track topography, a laser confocal microscope was used. The assessment of worn surface topography was estimated using eight parallel spots scanned with a 50× objective lens on each sample.

## 3. Results and Discussions

### 3.1. Microstructure

**Figure 3:**
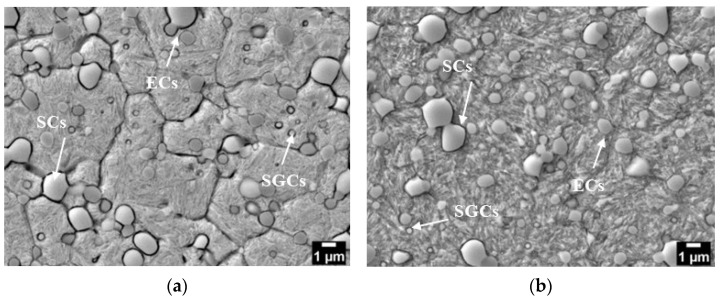
SEM microstructure of Vanadis 6 steel: (**a**) CT140170 and (**b**) CT140530.

SEM micrographs in Figure 3 display the microstructures of specimens subjected to cryogenic treatment at −140 °C and subsequent tempering at 170 °C (CT140170) and 530 °C (CT140530).

**Figure 4:**
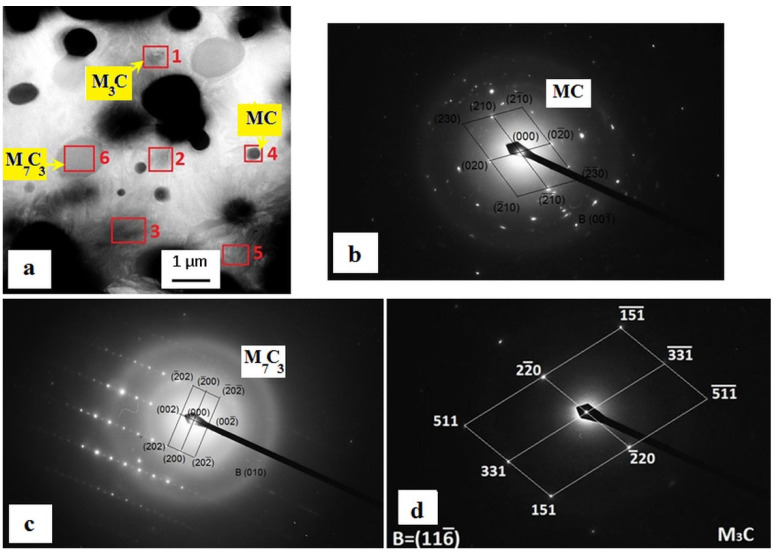
TEM micrograph (**a**) showing the microstructure of Vanadis 6 steel subjected to SZT for 17 h, and (**b**–**d**) diffraction patterns of MC, M_7_C_3_, and M_3_C carbides indicated in the micrograph. Adapted from recently published results on microstructural analyses of cryogenically treated Vanadis 6 steel [12].

**Figure 5:**
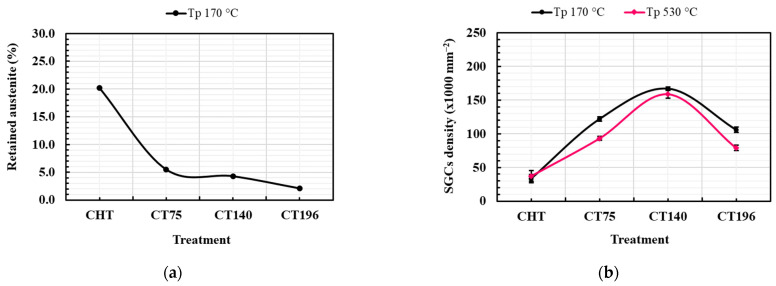
Results of quantitative analyses on: (**a**) retained austenite amount (%) in 170 °C tempered specimens (note that this analysis was not performed on 530 °C tempered specimens as this treatment removes the retained austenite almost completely from the steel microstructures) and (**b**) population density of (SGCs) (×10^−2^).

Both microstructures comprise martensite, some residual austenite (γ_R_) (although this phase is nearly eliminated in the CT140530 specimen), and three distinct types of carbides. Recently, thorough analyses of carbide phases in cryogenically treated Vanadis 6 steel were conducted using transmission electron microscopy (TEM) (Jeol Ltd., Tokyo, Japan) [12]. They revealed the prese nce of vanadium-based eutectic MC carbides (ECs), mixed chromium/iron-rich secondary carbides (SCs, M_7_C_3_), and small globular carbides (SGCs, M_3_C) in the steel after subjecting it to cryogenic treatments at −140 °C, as shown in Figure 4. Furthermore, the qualitative microstructural features identified are similar to those of other CTs and Tps used in this study; thus, only one CT micrograph is referenced here. The used treatment schedules do not alter the population densities of ECs and SCs, as previously reported [20,21,22], while varying the population density of SGCs. Figure 5 summarizes the amounts of γ_R_ and population densities of SGCs in the specimens subjected to different heat treatments.

When comparing the microstructure of specimens treated with cryogenic treatment (CT) and conventional treatment (CHT), two main observations can be made. First, the comparison of the amount of retained austenite in CT and CHT specimens shows that CT tends to reduce the γ_R_ content considerably. The CT temperature of −196 °C is the most effective at reducing the γ_R_ content among the CT temperatures used in this study, as shown in Figure 5a.

For example, the γ_R_ content for CHT specimens is around 20%, whereas it is reduced to only 2.1% after treating at −196 °C and tempered at 170 °C. Additionally, tempering at 530 °C nearly eliminated the retained austenite content for both CT and CHT specimens. Second, the diagram in Figure 5b demonstrates that the CT140170 specimen contains the maximum population density of SGCs. These findings are consistent with those of previous research on AISI D2 steel by Das et al. [13,26], who reported that CT at −196 °C eliminated the γ_R_ almost completely while enhancing the population density of carbides approximately threefold. The microstructure’s detailed explanatory features regarding different cryogenic treatments are not covered in this study, as they are already available in recent studies [20,21,22].

### 3.2. Hardness and Fracture Toughness (K_IC_)

**Figure 6:**
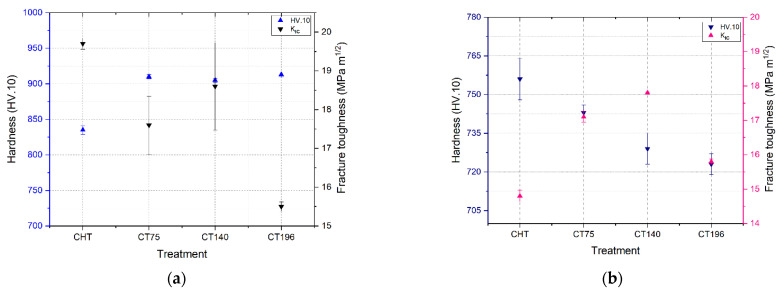
Hardness and fracture toughness (K_IC_): (**a**) samples tempered at 170 °C and (**b**) samples tempered at 530 °C.

The values of hardness and fracture toughness after CHT and different cryogenic treatments are shown in Figure 6.

The hardness is plotted on the primary *Y*-axis (blue columns) and the fracture toughness on the secondary *Y*-axis (black/pink columns). As shown in Figure 6a, it is evident that CT samples tempered at 170 °C exhibit a higher hardness, approximately 80 HV greater than CHT specimens. Although the hardness of CT specimens is higher than that of CHT, the difference in hardness between CT specimens shows insignificance, at a maximum of 10 HV. The hardness increase is estimated at around 8–9% over the CHT specimens. The augmentation in the hardness of tool steels through CT has been previously documented by various researchers in comparison to CHT, with variations noted in the degree of enhancement. For instance, Jun Li et al. [4] observed a 6% improvement in hardness for H13 tool steels following CT, while Das et al. [13,14] reported a 5% increase in bulk hardness for AISI D2 relative to CHT conditions. In both cases, the hardness improvement was attributed to the reduction of retained austenite and the increase in carbide count. Generally, this hardness increase is inversely proportional to fracture toughness (i.e., when hardness increases, toughness decreases); for instance, the fracture toughness of CHT170 specimens is 19.7 (MPa × m^1/2^), while for CT specimens CT75170, CT140170, and CT196170, it is 17.6, 18.6, and 15.5 (MPa × m^1/2^), respectively. The decrease in fracture toughness of CT specimens relative to CHT is around −10% for CT75170, −6% for CT140170, and −21% for CT196170. This trend underscores the optimal enhancement in hardness at only marginal fracture toughness reduction achieved with CT at −140 °C compared to other CT conditions. The decrease in fracture toughness attributed to CTs compared to CHT conditions has previously been documented in the literature concerning cold-work tool steels subjected to tempering at low temperatures. For instance, in the case of AISI D2 steel, a reduction in fracture toughness values of 9.9%, 21.1%, and 19.2% has been observed for cryogenic treatments at −75 °C, −125 °C, and −196 °C, respectively [27]. On the other hand, tempering at 530 °C (Figure 6b) shows a decrease in the hardness of CHT and CT specimens compared to the samples tempered at 170 °C and makes the hardness of CT specimens lower than that of CHT ones. But the fracture toughness of CT specimens was found to be higher than that of CHT specimens; for instance, the facture toughness of CHT specimens after tempering at 530 °C was 14.8 (MPa × m^1/2^), whereas for CT specimens, it was 17.1, 17.8, and 15.8 (MPa × m^1/2^), respectively, for CT75530, CT140530, and CT196530. This trend suggests that after high-temperature tempering, CT specimens showed 15%, 20%, and 6% increments in fracture toughness value over CHT specimens. These findings exhibit concordance with prior investigations conducted by researchers within the field. For instance, the impact toughness of AISI D6 steel following CT has been scrutinized by Cardoso et al. [28]. Their study revealed that subjecting the tool steel for a duration of 24 h at −180 °C resulted in a notable enhancement of impact toughness by 58.3% subsequent to tempering at 500 °C for 4 h and double tempering at 250 °C for 2 h. Microstructural changes play a pivotal role in these findings, with reduced retained austenite content in CT specimens contributing to increased hardness and decreased fracture toughness of steel tempered at 170 °C. But an increased small secondary carbide (SGC) count partly compensates for reduced fracture toughness, particularly evident for steel subjected to CT at −140 °C. After high-temperature tempering, the elimination of retained austenite and the gradual softening of the martensitic matrix with reduced SG carbide density are observed. However, even after high-temperature tempering, CT140 maintains higher K_IC_ values than other treatments, emphasizing its ability to improve material hardness and fracture toughness over other cryogenic temperatures and CHT treatments. This comprehensive analysis provides crucial insights for optimizing material treatments to achieve desired mechanical properties in practical applications.

**Figure 7:**
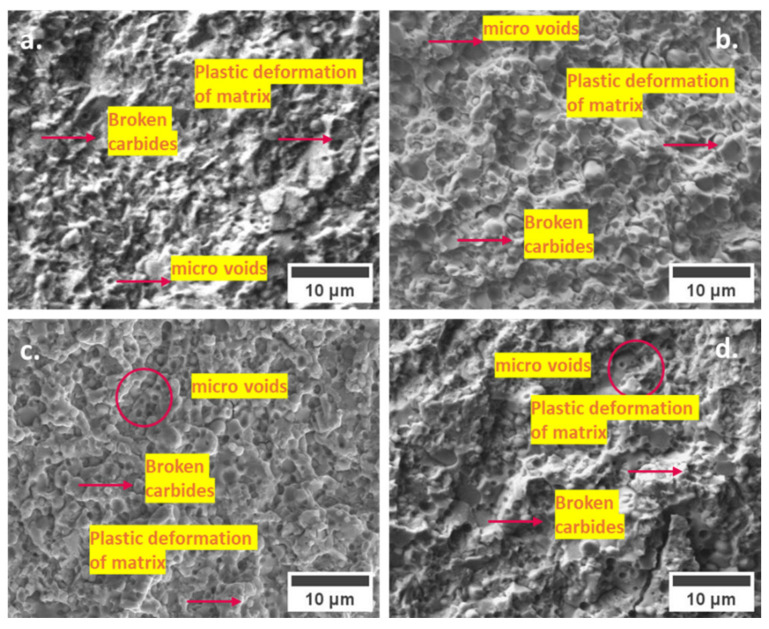
SEM microstructure of fracture surfaces of Vanadis 6 steel tempered at 170 °C: (**a**) CHT, (**b**) CT75, (**c**) CT140, and (**d**) CT196.

To comprehend the underlying fracture mechanisms, a comprehensive analysis was conducted on a series of images acquired from the fracture surfaces of all tested samples. As depicted in Figure 7, the obtained fractographs from the scanning electron microscope (SEM) unveil consistent failure patterns characterized by fractured coarser eutectic carbides, microplastic deformation of the matrix, and the emergence of microvoids originating from the matrix/carbide interface, particularly evident around secondary and smaller secondary carbides. Notably, the presence and distribution of these features on the fracture surfaces exhibit distinct variations across differently treated samples. For instance, CT at −140 °C showcases the most notable abundance of microvoids attributed to the decohesion of small secondary carbides (SGCs), consequently leading to the highest values of fracture toughness (K_IC_) compared to other CT specimens.

**Figure 8:**
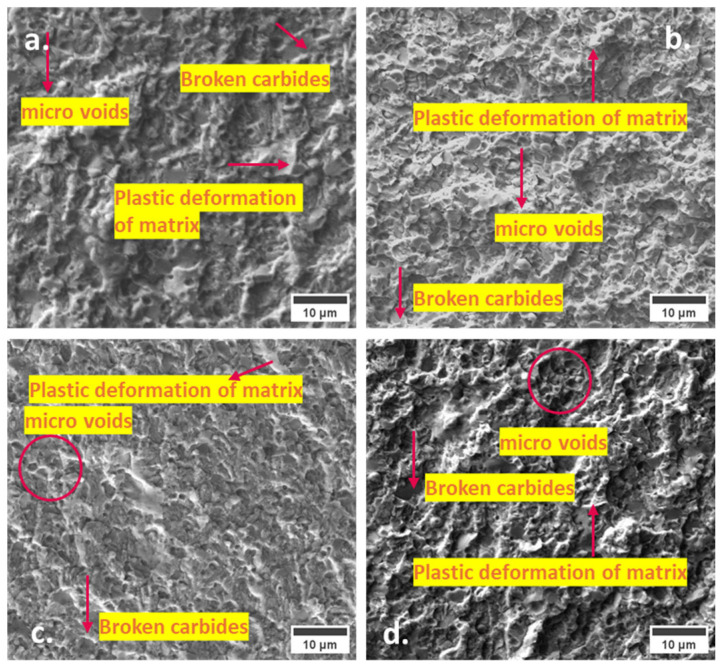
SEM microstructure of fracture surfaces of Vanadis 6 steel tempered at 530 °C: (**a**) CHT, (**b**) CT75, (**c**) CT140, and (**d**) CT196.

The fracture surfaces of specimens tempered at 530 °C exhibit notable distinctions compared to those tempered at 170 °C, as depicted in Figure 8. A limited occurrence of microvoids, along with localized plastic deformation of the matrix, is evident in all the specimens. It results in a flat appearance of fracture surfaces, which is most pronounced in CHT steel, as shown in Figure 8a. In contrast, the specimens subjected to CT retained a slightly higher number of microvoids, accompanied by more pronounced plastic deformation of the matrix. These observations underscore the disparities in fracture toughness (K_IC_ values) between CT and CHT specimens.

**Figure 9:**
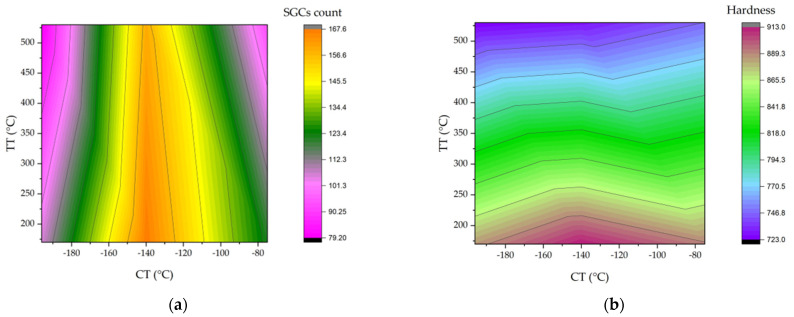
Contour plots: (**a**) treatment vs. small globular carbides (×1000^−2^) and (**b**) treatment vs. hardness.

Figure 9 illustrates the correlations between cryogenic and tempering treatments and material properties.

The contour plots show that the material achieves its highest hardness and small globular carbide densities at a cryogenic treatment temperature of −140 °C. This intriguingly coincides with the optimum observed fracture toughness values at the same temperature. This suggests a strong link between cryogenic treatment at −140 °C, improved hardness, increased small secondary carbide density, and enhanced fracture toughness, as shown in Figure 6b.

### 3.3. Coefficient of Friction (COF)

**Figure 10:**
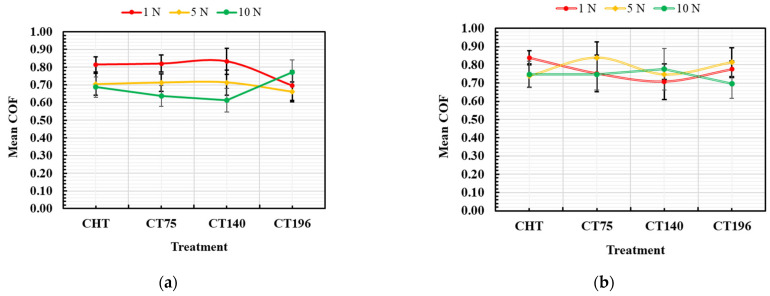
Mean coefficient of friction as a function of load for different treatments: (**a**) tempered at 170 °C and (**b**) tempered at 530 °C.

Figure 10 illustrates the mean coefficient of friction (COF) values, revealing significant trends associated with different tempering temperatures and treatment conditions.

Notably, specimens tempered at 170 °C consistently exhibit slightly lower COF values compared to those tempered at 530 °C. Another key observation is the inverse relationship between load and COF, particularly evident in low-temperature tempered specimens, where higher loads lead to lower COF values. This aligns with findings from prior studies by Podgornik et al. [29] and Korade and Ramana [18], emphasizing the impact of high-speed steel hardness on COF reduction after cryogenic treatment. They claimed that the reduction in COF can be attributed to the higher hardness of evaluated high-speed steels or AISI D2 material in testing against WC counterparts. Moreover, Korade and Ramana [18] proposed that the reduction of soft retained austenite, along with a higher number and better distribution of fine carbides, could be responsible for the reduction of COF. In the present study, it was found that specimens tempered at 170 °C manifested the best combination of retained austenite reduction and enhanced number of carbides, resulting in lower COF values. Notably, the increased number of carbides effectively lowers the COF value at more severe contacting conditions, at 10 N applied load, for instance. The influence of loading severity on COF was investigated by Jovičevič-Klug and Podgornik [30]. They also found that increasing load leads to lower COF.

**Figure 11:**
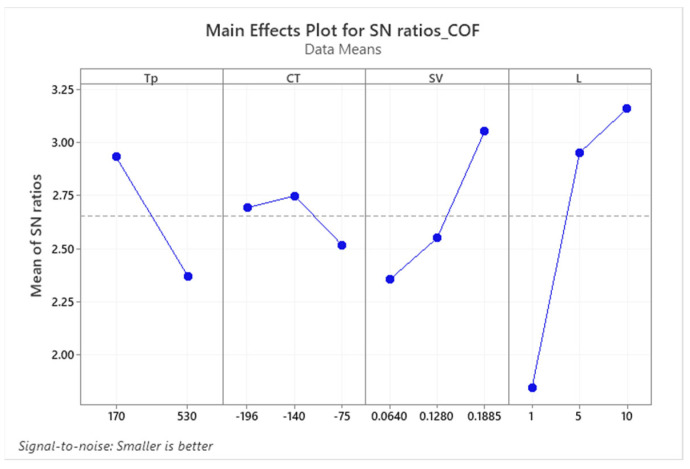
Main effects plot for SN ratios of coefficient of friction (COF).

Utilizing the Taguchi design method, the main effects plot in Figure 11 and the corresponding ANOVA results in Table 2 identify optimal conditions for minimizing COF as: tempering at 170 °C, cryogenic treatment at −140 °C, sliding speed of 0.1885 m/s, and a load of 10 N. Statistical analysis underscores the predominant influence of load (56.7%), sliding velocity (14.7%), and tempering temperature (13.5%) on COF, with a marginal impact from cryogenic treatment temperature (1.7%). The possible reason for the marginal impact of cryogenic treatment could be a result of no significant difference in the hardness of different cryogenic treatments, as shown in Figure 6. This strengthens the claim that COF depends mainly on the load, then on the sliding velocity, and also on the tempering temperature in the examined conditions.

**Figure 12:**
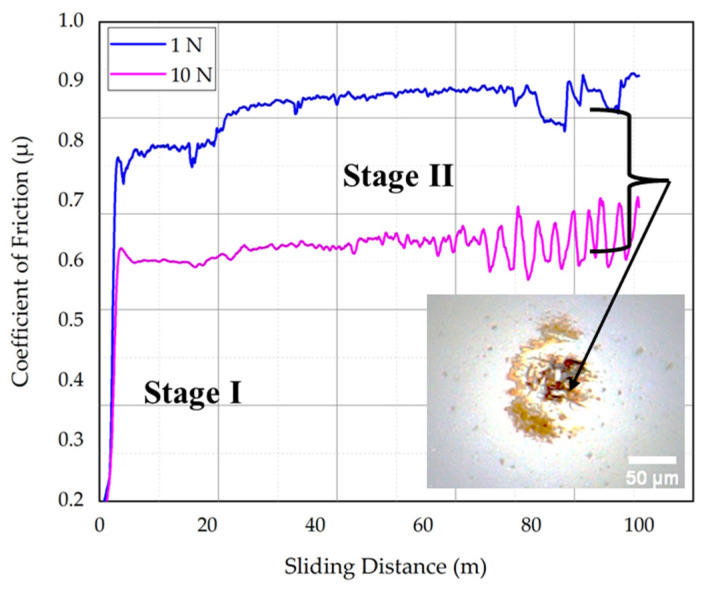
Dependence of COF on sliding distance for the CT140170 sample tested under 10 N loading and for the CT75530 sample tested under 1 N load.

Figure 12 illustrates the sliding distance dependence of the COF values for two representative specimens: CT140170 tested at 10 N (optimum condition) and CT75530 tested at 1 N (worst condition).

The study revealed varying COF values for cryogenically treated Vanadis 6 ledeburitic tool steel. The best results were obtained with CT140170 at 10 N, yielding a steady-state COF of 0.61 ± 0.1. Conversely, the worst-performing CT75530 trial resulted in a steady-state COF of 0.88 ± 0.05. The inset in Figure 12 highlights that the significantly harder alumina counterpart contributes to surface irregularity destruction, generating a tribo-oxidative coating on both surfaces. This coating undergoes repeated removal and adhesion during sliding, leading to COF fluctuations, particularly within the 70–100 m sliding distance range. Sliding velocity significantly influences COF values, as depicted in Figure 11. Higher velocities correlate with lower COF values, possibly due to increased work hardening of wear debris and enhanced oxidation. This effect is consistent with prior studies on AISI D2 steel [16], where higher sliding speeds yielded thicker surface oxides and thereby reduced COF values. In summary, the applied load, sliding velocity, and tempering treatment all influence COF, with cryogenic treatment at −140 °C demonstrating superior tribological performance.

### 3.4. Volume Loss (V)

**Figure 13:**
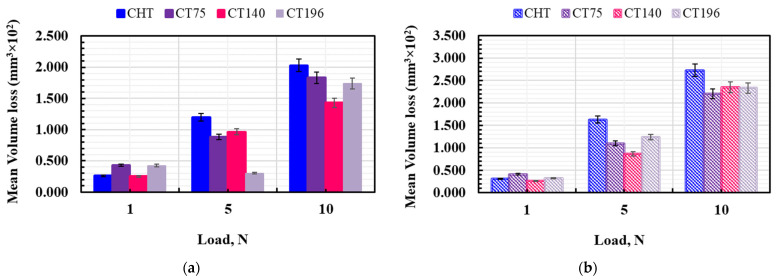
Volume loss (V) of material as a function of load: (**a**) tempered at 170 °C and (**b**) tempered at 530 °C.

Figure 13 presents the volume loss (mm^3^), including reference data for conventionally heat-treated (CHT) specimens. The first trend in the obtained results is that the specimens tempered at lower temperatures exhibit lower volume loss, indicating improved wear resistance compared to those tempered at higher temperatures. Furthermore, the volume loss consistently increases with the applied load across all treatment conditions. This aligns with findings by other researchers studying cold-work tool steel [15].

Furthermore, the charts demonstrate that, under increasingly severe tribological conditions, cryogenic treatments result in lower volume losses compared to CHT specimens. Within the cryogenic treatments, −140 °C cryogenic treatment, followed by low-temperature tempering, yields the lowest volume loss compared to other treatment routes used in the present study, as shown in Figure 13a. This processing approach, therefore, holds significant potential for enhancing the wear performance of Vanadis 6 steel. For instance, at low-temperature tempering, CT140 treatment demonstrates −3%, −20%, and −30% lower volume loss compared to CHT specimens at 1 N, 5 N, and 10 N applied loads, respectively. When tempered at 530 °C, cryogenic treatments also led to a lowering of volume loss, but the effect of CT temperature is insignificant at 10 N load, as shown in Figure 13b.

**Figure 14:**
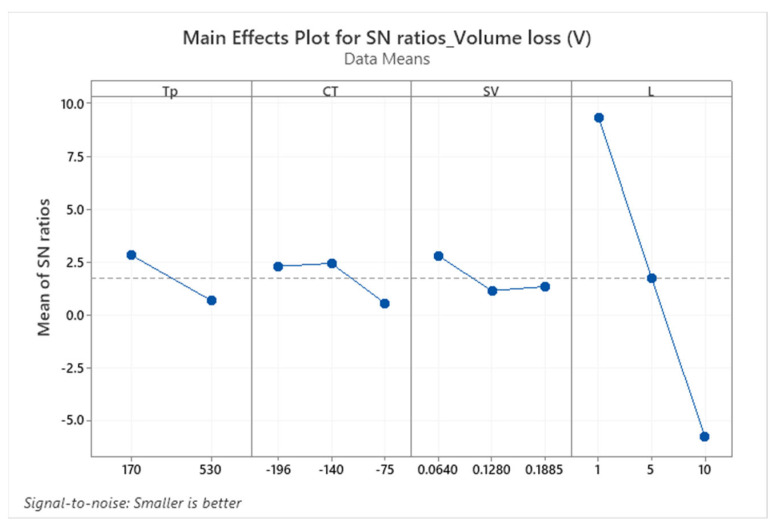
Main effects plot for SN rations of volume loss (V).

In Figure 14, the significant impact of applied load on volume loss is evident, with increasing load leading to higher volume loss.

Similarly, tempering temperature also showed a linear negative relationship to volume loss, representing higher-tempering temperatures leading to higher volume loss. The statistical analysis presented in Table 3 highlights load as a significant factor, contributing 84% to explaining the volume loss of the material. However, both cryogenic treatment and sliding velocity have a non-linear relationship with volume loss, and treatment at −140 °C in combination with lower sliding velocities is the optimum setting to improve the tribological performance of the examined steel.

In line with earlier research emphasizing the benefits of cryogenic treatment on wear resistance in D-class steels [13,14,15,19,31,32,33], this study confirms that optimal hardness and a maximum number of extra carbides were achieved after cryogenic treatment at −140 °C, as shown in Figure 5b. This finding aligns with improved wear performance, indicating a correlation between microstructural changes, mechanical properties, and tribological behavior. The increased carbide counts and the reduction of retained austenite (γ_R_), consistent with the literature [13,14,15,18,31,34], contribute to enhanced hardness during low-temperature tempering. The importance of the presence of hard carbides in the material microstructures with respect to their wear resistance can also be demonstrated based on the results by Arsič et al. [35]. They hardfaced a low-alloy steel with different materials, whose very similar hardness ranged between 56 and 58 HRC. However, the 4%C-19%Cr-4%W alloy outperformed all the other alloys, with much lower carbide amounts despite comparable bulk hardness. Many examples of the beneficial effects of carbide phases have been reported in one of the most recent review papers [36]. At this place, it should be noted that, in 1994, Meng et al. [37] observed that cryogenic treatments foster the precipitation of transient η-carbides at low tempering temperatures. They have examined, among others, the wear resistance of cryogenically treated AISI D2 steel and claimed that a higher amount and better homogeneity of nano-sized η-carbides may be responsible for the better wear performance of cryogenically treated material. The assumption of fostered precipitation due to cryogenic treatments was more recently reaffirmed for the Vanadis 6 steel [11,20]. One can expect that these coherent precipitates increase both the matrix hardness and its stiffness and thereby contribute to the better abrasive wear performance of CT steels. And finally, certain effects of fracture toughness on wear performance can also be expected. Of note, the −140 °C treated steel has the highest fracture toughness among cryogenically treated specimens, and its wear performance is the best. At the highest applied load, for instance, one can hypothesize that better fracture toughness may make the material more resistant against crack propagation in the plastically deformed near-to-surface region, resulting in lower volume loss.

Previous research has established that subjecting D-class steels to high-temperature tempering subsequent to cryogenic treatment yields negligible improvements in hardness [19]. In such instances, the microstructures of the steel lack γ_R_, and while the abundance of additional carbide particles diminishes compared to pre-tempered states, it remains elevated relative to conventionally heat-treated (CHT) steels [20,21,22]. Nevertheless, the matrix of CT steels experiences more extensive softening in contrast to conventionally treated materials, as evidenced by earlier investigations [20,22]. Initial investigations failed to discern any significant influence of cryogenic treatment on wear resistance against alumina counterparts [38]. However, this study employed a −196 °C cryogenic treatment (for only 4 h) followed by tempering at 530 °C. The present findings suggest that treatment at −140 °C may confer certain enhancements in wear resistance compared to treatment at −196 °C when tested under 1 N and 5 N loads, while demonstrating comparable performance under 10 N loads. Nevertheless, when compared with the CHT state, all cryogenic treatments exhibit better wear performance with increasing severity of contact conditions. At this place, it should be mentioned that Leskovšek, Kalin, and Vižintin [39] suggested a positive effect of higher fracture toughness on the wear performance. They treated cryogenically (at −196 °C for 1 h) the AISI M2 high-speed steel and found that only a slight improvement in the fracture toughness led to a significant increase in the wear resistance. This coincides with the results obtained in the current study, where better wear performance of cryogenically treated and high-temperature tempered samples has been recorded as compared with CHT samples.

### 3.5. Worn Surface Characterization

**Figure 15:**
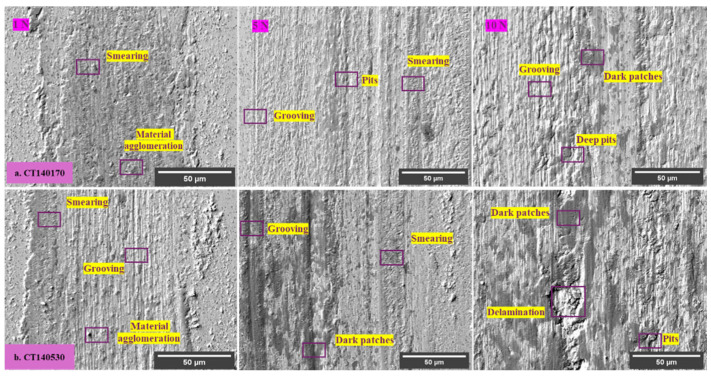
SEM micrographs of worn-out surfaces after tempering at 170 °C and 530 °C.

Figure 15 displays scanning electron (SEM) micrographs illustrating the worn surfaces of CT140 specimens post-tempering at 170 °C and 530 °C under varying tribological conditions. Irrespective of the treatment strategy or testing conditions employed, the worn surfaces consistently manifested a rough, shiny, and metallic appearance. Distinctive features included parallel grooves aligned with the sliding direction, alongside pits of varying depths. Furthermore, the SEM analysis revealed the presence of smeared steel material in the form of agglomerates, predominantly situated along the sides of all wear tracks, along with occasional dark patches.

The extent of surface damage that is characterized by the presence of deep pits, grooves, and dark patches generally increases with the increase in normal load, sliding speed, and tempering temperature. For the testing under a constant load, the surface damage increases in the order of specimens processed at −140 °C, −75 °C, and −196 °C, respectively. The worn surfaces of 530 °C tempered samples, in addition, manifest symptoms of surface delamination after testing under a 10 N load. The same applies to the −75 °C-treated samples when tested at 5 or 10 N. The wear severity is thus the lowest for −140 °C steel samples, and it is more extensive for other cryogenic treatments applied in the present study. This is also in line with the data in Figure 13, where the specimens treated at −140 °C manifest the lowest volume loss in most cases of testing conditions; thus, they outperform the −75 and −196 °C treated ones, despite the latter also showing improved wear performance as compared with CHT steel.

**Figure 16:**
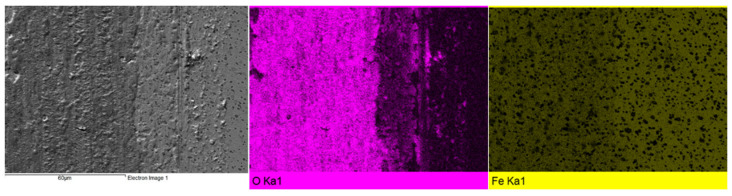
SEM−EDS maps of oxygen and iron in the wear track of the CT140170 sample developed by the dry sliding of alumina under a load of 1 N.

High Hertzian pressures make it possible to transfer the removed wear debris to the sides of wear tracks during sliding. At these sites, the wear debris is subjected to plastic deformation, which increases its susceptibility to oxidation. SEM micrographs and EDS maps in Figure 16 provide clear evidence of extensive oxidation of transferred wear debris upon an example of the worn surface of the CT140170 specimen after testing under a load of 1 N. On the left side of the micrographs and EDS maps, there is the agglomerated material, which manifests symptoms of extensive oxidation. Of note, similar tribo-oxidative wear modes have been determined, for instance, in the case of boronized AISI 316L steel when tested against hard ceramics and under similar load-sliding speed circumstances [40,41].

Almost no evidence of oxide layers was identified when higher sliding speeds were used, except in some cases of 530 °C tempered steel. Instead, deep grooves, extensive abrasion, and occasional pitting were observed on worn surfaces. The severity of surface damage increased with load and is more pronounced in the case of high-temperature (530 °C) tempered samples, as shown in Figure 15. The lower material hardness of the 530 °C tempered steel allowed for greater plastic deformation, leading to extensive oxidation of the plastically deformed material. The dark appearance of the patches on the worn surfaces is attributed to the presence of high levels of low-atomic oxygen. It is worth noting that a special “mixed” detection regime, consisting of 50% secondary and 50% backscattered electrons, was used to detect both the surface topography and the presence of oxidic layers on metallic surfaces.

**Figure 17:**
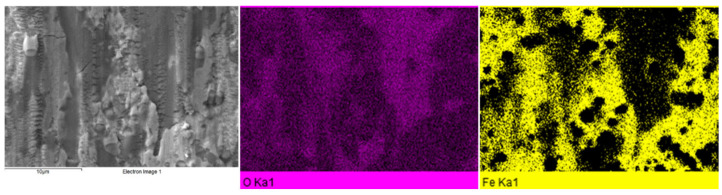
SEM−EDS maps of oxygen and iron of the wear track of the CT140170 sample developed by the dry sliding of alumina under a load of 10 N.

Plan-view SEM micrograph of the worn surface of the CT140170 sample after testing under a 10 N load, and corresponding EDS maps for oxygen and iron indicate that dark patches coincide with the sites of high oxygen content, as shown in Figure 17. This finding strengthens the claim about the higher susceptibility of plastically deformed steel to oxidation. Also, it is in excellent agreement with recently published articles that were focused on the investigation of the abrasive wear performance of cryogenically treated AISI D2 tool steel. [15,42]. The authors of the cited studies discovered that extensive plastic deformation caused by frictional shear stress fosters the formation of a tribo-oxide layer. They also observed that the oxide layer thickness tends to increase with increasing load and lower steel hardness. In the present study, tempering at 530 °C made the steel softer than tempering at 170 °C, irrespective of the cryogenic treatment applied. Mentioned softening results in a more extensive occurrence of pitting and tribo-oxidation of high-temperature tempered specimens, as compared in Figure 15 and Figure 17.

**Figure 18:**
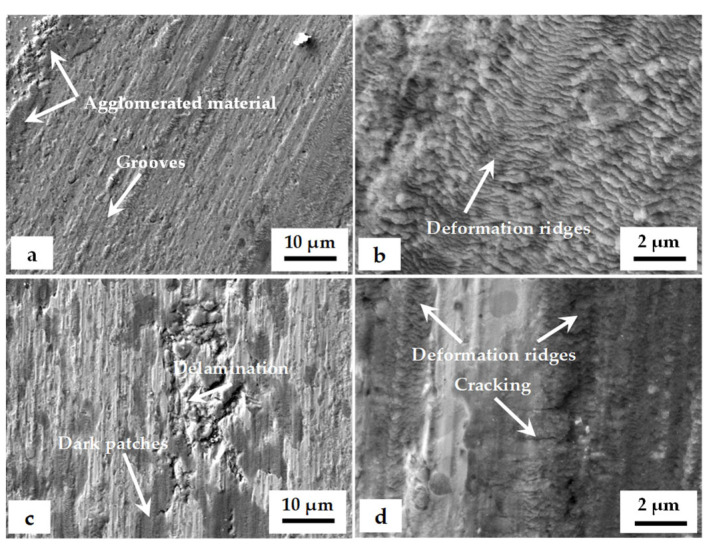
SEM images of wear tracks of CT196530 samples: (**a**) at 1 N, (**b**) at 1 N detailed image of deformation ridges, (**c**) at 10 N, and (**d**) at 10 N detailed view of microcracking.

In some cases, deformation ridges were detected within the wear tracks. This phenomenon is typical, for instance, for the CT140170 specimen, as shown in Figure 16 and Figure 17. Also, in the case of the CT196530 specimen, the presence of these ridges was established, but only when tested at low load, as shown in Figure 18a,b. On the other hand, the wear was realized by deep and extensive grooving, as was the case when the latter sample was tested under a 10 N load. This may be explained by the following mechanism: under severe load and relative motion of the sample and counterpart wear debris, particles are formed. They can be easily oxidized and act as third-body abrasives, plowing the sample material out and producing grooves of different depths [43]. One can also assume that this wear mechanism is more active in the case of 530 °C tempered specimens since they have generally lower hardness as compared with the 170 °C tempered ones.

The wear tracks of the CT196530 sample that was tested under a 10 N load manifested a great number of dark patches. Also, clear indications of surface delamination and the presence of deep pits were observed, as shown in Figure 18c. A detailed SEM image in Figure 18d reveals that surface delamination takes place only after the plastic deformability of the close-to-surface material is exhausted. In other words, the material is first deformed plastically, then the first microcracks are formed, and, after their accumulation, the deformed material is removed from the surface.

**Figure 19:**
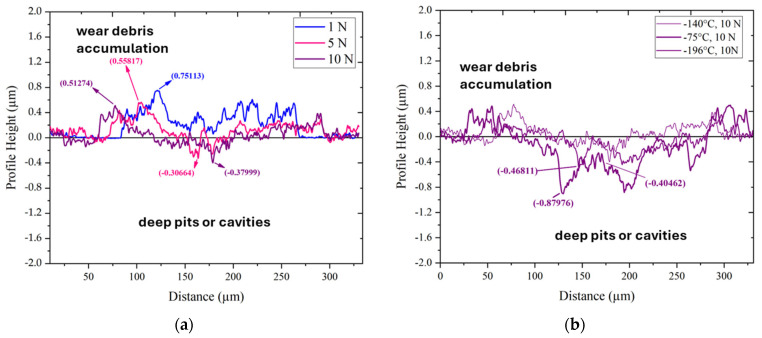
Topographic details of worn surfaces of the CT140170 specimen acquired by confocal microscopy: (**a**) wear profiles as a function of applied load and (**b**) wear profiles as a function of applied treatments at 10 N.

Images depicting the surface topography within the wear tracks developed under different normal loads are shown in Figure 19. The average surface roughness (Ra) tends to decrease with increasing load, as shown in Figure 19a. This may be explained by the logical assumption that under higher loads, local asperities on the surface are more effectively smoothed than expected under lower loads.

Cross-sectional profiles of wear tracks of differently treated samples tested under a 10 N loading are shown in Figure 19b. It is seen that the application of CT140170 treatment results in a smoother cross-sectional profile than other applied strategies in the current study. The maximum depth of the developed pit, for instance, was 0.345, 0.512, and 0.880 µm for the wear tracks developed on the CT140170, CT75170, and CT196170 specimens. These results also support the fact that the treatment strategy that involves the −140 °C cryogenic treatment followed by low-temperature tempering is the most promising way to minimize the abrasive wear of the examined steel.

Table 4 summarizes the operative wear mechanisms and modes for all the heat treatment strategies and testing conditions used in the present study. Both the wear mechanism and mode manifest clear variations, mainly depending on the applied load. At lower loads, mild tribo-oxidation was the most pronounced mechanism, but severe delaminative mechanisms were prevalent at high loads. However, it is also seen that the threshold of the wear mechanism change lies at higher loads for CT140170 samples, suggesting that this treatment strategy provides the experimental steel with the best wear performance.

The results obtained in the present study are in good agreement with those by Das et al. [15]. These investigators have examined the wear performance of cryogenically treated AISI D2 steel under different loads and sliding speeds. They discovered that conventionally heat-treated specimens manifest severe delaminative wear, irrespective of the sliding speeds used. But the application of −196 °C cryogenic treatment shifts the wear mechanism towards a mild oxidative one under specific testing conditions. In other publications [14,44], the same investigators have conducted exhaustive investigations in order to establish the transition load (a load where the mechanism changes from mild to severe) for different CT specimens made of AISI D2 steel. The principal finding was that CT at −196 °C significantly increased the transition load, which was referred to as almost complete removal of retained austenite, an enhanced number of hard carbides and their better distribution, and thereby a higher bulk hardness. In the current study, the application of CT at −140 followed by low-temperature tempering induces the best combination of retained austenite reduction and the extent of carbide count increase; hence, the highest transition load and sliding speeds are logically to be expected for the material treated in such a way.

## 4. Conclusions

In this study, the impact of different cryogenic treatment routes, specifically at −75 °C, −140 °C, and −196 °C, following low-temperature and high-temperature tempering treatments, on the hardness, fracture toughness, and abrasive wear behavior of Vanadis 6 tool steel against its alumina counterpart was investigated. The material properties were then correlated with microstructures and hardness values.

Cryogenic treatments resulted in a notable 8–9% increase in hardness compared to conventional treatment when tempered at 170 °C, while tempering at 530 °C led to a 3% decrease in hardness.

Cryotreated specimens exhibited an average of 15% improved fracture toughness after tempering at 530 °C compared to conventional treatment, contrasting with an average 12% decreased fracture toughness when tempered at 170 °C. Notably, CT at −140 °C showcased the optimal combination of hardness and fracture toughness in both low- and high-temperature tempering conditions.

Results indicated that specimens tempered at 170 °C generally displayed lower coefficients of friction than those tempered at 530 °C. Additionally, lower loads yielded higher coefficients of friction compared to higher loads. Cryogenic treatment at −140 °C in combination with higher sliding speeds and greater applied loads resulted in a minimum friction coefficient.

Furthermore, wear mechanisms were analyzed at different loads. At a nominal load of 1 N, the prevailing wear mechanism exhibited mild tribo-oxidation. With an increase in load to 5 N, a combination of tribo-oxidation and mild delamination became evident. Under more demanding tribological conditions, particularly at a load of 10 N, the wear mechanism transitioned to severe delamination.

## Figures and Tables

**Table 1:** Nominal and measured chemical compositions of the examined steel.

Chemical Composition (wt.%)	C	Si	Mn	Cr	Mo	V	Fe
Nominal	2.1	1.0	0.4	6.8	1.5	5.4	Bal.
Measured	1.98	1.05	0.42	6.82	1.47	5.02	Bal.

**Table 2:** Analysis of variance (ANOVA) table of coefficient of friction (COF).

Source	DF	Seq SS	Adj SS	Adj MS	F	P	%C
Tp	1	1.419	1.419	1.419	10.10	0.010	13.47
CT	2	0.177	0.177	0.088	0.63	0.552	1.68
SV	2	1.553	1.553	0.776	5.53	0.024	14.75
L	2	5.978	5.978	2.989	21.27	0.000	56.75
Residual error	10	1.405	1.405	0.140			
Total	17	10.533					

Tp: tempering temperature, CT: cryogenic temperature, SV: sliding velocity, L: load, DF: degrees of freedom, Seq SS: sequential sum of squares, Adj SS: adjusted sum of squares, Adj MS: adjusted mean squares, F: f-statistic, P: probability, %C: contribution.

**Table 3:** Analysis of variance (ANOVA) table of volume loss (V).

Source	DF	Seq SS	Adj SS	Adj MS	F	P	%C
Tp	1	20.963	20.963	20.963	2.33	0.158	3%
CT	2	13.332	13.332	6.666	0.74	0.501	2%
SV	2	9.843	9.843	4.922	0.55	0.595	1%
L	2	686.335	686.335	343.167	38.1	0	84%
Residual error	10	90.059	90.059	9.006			
Total	17	820.531					

Tp: tempering temperature, CT: cryogenic temperature, SV: sliding velocity, L: load, DF: degrees of freedom, Seq SS: sequential sum of squares, Adj SS: adjusted sum of squares, Adj MS: adjusted mean squares, F: f-statistic, P: probability, %C: contribution.

**Table 4:** Operating wear mechanisms and modes for the experiments.

Cryogenic Treatment	Load, N	Wear Mechanism	Mode
CT75170	1	Tribo-oxidative	Mild
CT75170	5	Delaminative	Severe
CT75170	10	Delaminative	Severe
CT140170	1	Tribo-oxidative	Mild
CT140170	5	Mixed	Moderate
CT140170	10	Delaminative	Severe
CT1196170	1	Tribo-oxidative	Mild
CT196170	5	Mixed	Moderate
CT196170	10	Delaminative	Severe
CT75530	1	Tribo-oxidative	Mild
CT75530	5	Delaminative	Severe
CT75530	10	Delaminative	Severe
CT140530	1	Tribo-oxidative	Mild
CT140530	5	Delaminative	Severe
CT140530	10	Delaminative	Severe
CT196530	1	Tribo-oxidative	Mild
CT196530	5	Delaminative	Severe
CT196530	10	Delaminative	Severe

## Data Availability

The data are available upon a request.
